# Enhancing human ACE2 expression in mouse models to improve COVID‐19 research

**DOI:** 10.1002/2211-5463.13934

**Published:** 2024-12-29

**Authors:** Sun Jiaoyang, Cheng Shaofei, Hong Guangliang, Quan Xiongzhi, Lin Haofeng, Mao Rui, Johannes Grillari, Shi Zheng‐Li, Chen Jiekai, Liu Meiqin, Wu Haoyu, Wu Guangming

**Affiliations:** ^1^ Center for Cell Lineage and Development, Guangzhou Institutes of Biomedicine and Health Chinese Academy of Sciences Guangzhou China; ^2^ University of Chinese Academy of Sciences Beijing China; ^3^ Guangzhou National Laboratory Guangzhou China; ^4^ Wuhan Institute of Virology Chinese Academy of Sciences Wuhan China; ^5^ Laboratory Animal Research Centre, Guangzhou Institutes of Biomedicine and Health Chinese Academy of Sciences Guangzhou China; ^6^ Austrian Cluster for Tissue Regeneration Vienna Austria; ^7^ Institute of Molecular Biotechnology BOKU University Vienna Austria; ^8^ Ludwig Boltzmann Institute for Traumatology The Research Center in Cooperation with AUVA Vienna Austria; ^9^ Guangdong Provincial Key Laboratory of Stem Cell and Regenerative Medicine, Guangdong‐Hong Kong Joint Laboratory for Stem Cell and Regenerative Medicine, Guangzhou Institutes of Biomedicine and Health Chinese Academy of Sciences Guangzhou China; ^10^ Centre for Regenerative Medicine and Health, Hong Kong lnstitute of Science & Innovation Chinese Academy of Sciences Hong Kong SAR China; ^11^ The First Affiliated Hospital of Guangzhou Medical University, Guangzhou Laboratory Clinical Base Guangzhou Medical University Guangzhou China; ^12^ The Third Affiliated Hospital of Guangzhou Medical University Guangzhou China; ^13^ Present address: Guangzhou National Laboratory Guangzhou China

**Keywords:** COVID‐19, hACE2, mouse model, β‐globin

## Abstract

Mice are one of the most common biological models for laboratory use. However, wild‐type mice are not susceptible to COVID‐19 infection due to the low affinity of mouse ACE2, the entry protein for SARS‐CoV‐2. Although mice with human ACE2 (hACE2) driven by *Ace2* promoter reflect its tissue specificity, these animals exhibit low ACE2 expression, potentially limiting their fidelity in mimicking COVID‐19 manifestations and their utility in viral studies. Here, we created and compared hACE2 mouse models generated with different strategies. Our findings show that distinct β‐globin insertion within hACE2 cassette significantly influences its expression, with downstream placement enhancing transcription. Moreover, optimizing hACE2 codons (opt‐hACE2) improves translation efficiency in multiple tissues. Notably, opt‐hACE2 mice displayed more active immune responses and severe COVID‐19 phenotypes following SARS‐CoV‐2 challenge compared to other models. Our study demonstrates the dual regulatory role of β‐globin element in transgene transcription and suggests that opt‐hACE2 mice might serve as valuable tools for SARS‐CoV‐2 research.

## Graphical abstract

We developed an enhanced hACE2 mouse model by incorporating the β‐globin sequence downstream of *ACE2* and optimizing ACE2 codon usage. These optimized hACE2 (opt‐hACE2) mice exhibited higher hACE2 expression across multiple tissues and demonstrated more severe COVID‐19 symptoms following SARS‐CoV‐2 challenge. Our studies highlight opt‐hACE2 mice as valuable tools for COVID‐19 research.

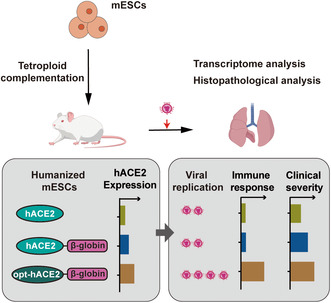

**Abbreviations**
COVID‐19: coronavirus disease 2019
hACE2: human angiotensin‐converting enzyme 2
SARS‐CoV‐2: severe acute respiratory syndrome coronavirus 2
WPRE: woodchuck hepatitis virus (WHP) posttranscriptional regulatory element

The worldwide spread of severe acute respiratory syndrome coronavirus 2 (SARS‐CoV‐2), causing over 600 million infections and 6.8 million deaths, has severely threatened the physical and mental health of human beings since 2019 (https://coronavirus.jhu.edu/map.html). Although the development of vaccines and antiviral drugs has slowed down the transmission and reduced the severity of the disease, the precise mechanisms underlying SARS‐CoV‐2 pathogenesis remain incompletely understood. Moreover, the new upcoming SARS‐CoV‐2 variants, for example, the Delta and Omicron strains, present ongoing challenges and are to be further explored for their infectiousness and pathogenicity [1]. Thus, investigation of SARS‐CoV‐2 as well as its mutant strains remains crucial for the improvement of vaccination and the development of novel antiviral therapies for coronavirus disease 2019 (COVID‐19).

Animal models are invaluable resources for virus infection study, with mice serving as a well‐established biological model for laboratory use. However, wild‐type mice are not susceptible to COVID‐19 infection due to the low affinity of mouse ACE2, the entry protein for SARS‐CoV‐2 [2]. To address this natural limitation, various strategies have been developed, including introducing human ACE2 (hACE2) into mice or creating mouse‐adapted virus strains [3]. For example, transient expression of human ACE2 via adeno‐associated virus (AAV) delivery to the respiratory tract enables mice to be infected by SARS‐CoV‐2. While infected mice display viral replication, antibody production, and inflammation in the lungs, potential impacts on other ACE2‐expressing tissues may be overlooked due to the limited area of ACE2 delivery [4]. K18‐humanized ACE2 (hACE2) mice, in which *ACE2* is driven by cytokeratin‐18 (K18) promoter, were widely used for SARS‐CoV studies [5]. They display typical clinical symptoms and produce high viral loads upon SARS‐CoV‐2 infection. As K18‐hACE2 transgenic mice also exhibit pathogenic changes from other tissue that are normally not found in COVID‐19 patients, other strategies were developed in which *ACE2* is driven by more specific promoters [3]. One approach involves inserting human *ACE2* into the mouse *Ace2* locus, thereby driving its expression using the mouse *Ace2* promoter [6]. This manner allows for tissue‐specific expression of human ACE2 in mice, offering a more accurate model for studying the natural pathological manifestations of COVID‐19 in humans.

Previously, we reported the generation of hACE2 mice within 35 days using stem‐cell‐based editing combined with tetraploid complementation techniques [7]. Our mice demonstrated body weight loss, virus replication, and lung inflammation following SARS‐CoV‐2 infection [7]. Here, we reported further refinement of our hACE2 expressing system by the β‐globin element incorporation and codon optimization. Our results suggest that the addition of β‐globin exerts a local‐specific bifunctional effect on hACE2 transcription, while optimizing codon usage enhances translation efficiency and protein production. Upon SARS‐CoV‐2 infection, opt‐hACE2 mice exhibited a more active immune response compared to the other tested hACE2 mice models. Thus, we concluded that our opt‐hACE2 mouse model could serve as a better mouse model for COVID‐19 studies.

## Materials and methods

### Experimental animals

Wild‐type Balb/c mice were purchased from the Model Animal Research Center of Nanjing University. Mice were maintained under specific‐pathogen‐free (SPF) conditions with a 12‐h light/dark cycle (illuminated from 7:00) and granted unrestricted access to both food and water. These mice were used when they were 6–10 weeks old. All the animal experiments in this study were approved by The Institutional Animal Care and Use in Guangzhou Institute of Biomedicine and Health (GIBH). The approved animal ethical and welfare number is IACUC‐2020120. All animal care and handling procedures were performed in accordance with ARRIVE guidelines. All the procedures involving animal works are complied with all relevant ethical regulations.

### Cell lines

Balb/c mouse embryonic stem cells (ESCs) were derived from 3.5 d.p.c inner cell mass (ICM) from Balb/c female mice crossed with Balb/c male mice. Mouse ESCs were cultured on feeders in DMEM containing 15% fetal bovine serum, 1000 U·mL^−1^ LIF, MEK inhibitor PD0325901 (1 mm), and GSK3 inhibitor CHIR99021 (3 mm).

### Generation of hACE2 mouse ESCs

**Fig. 1:**
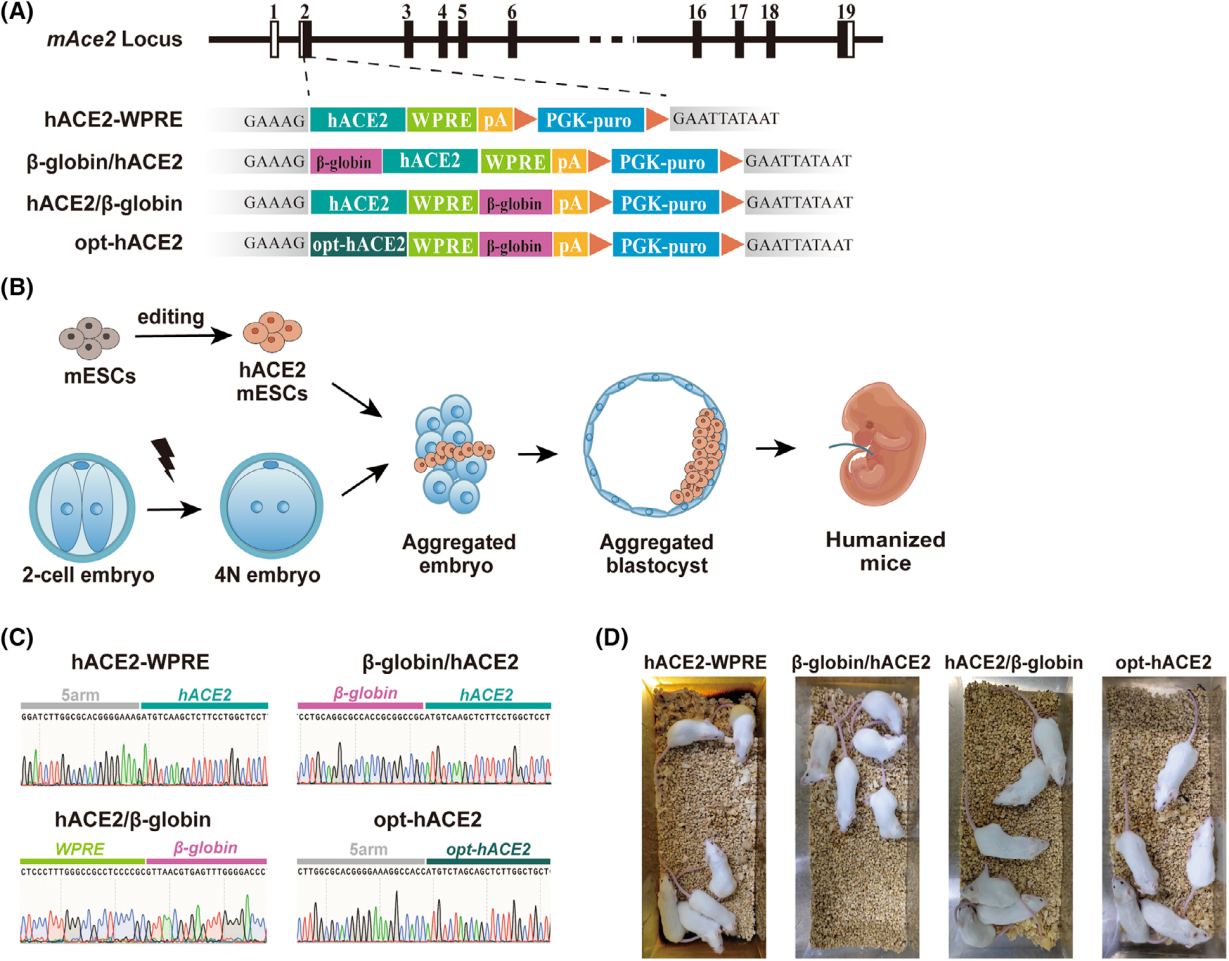
Characterization of different hACE2 mouse models. (A) Schematic diagram showing four different molecular cloning strategies including WPRE, β‐globin/hACE2 (upstream), hACE2/β‐globin (downstream), and opt‐hACE2 for *hACE2* cassette insertion at the *mAce2* locus. (B) Schematic diagram of different hACE2 mice generated using mES editing combined with tetraploid complementation techniques. (C) Sanger sequencing results at the key positions of the hACE2 cassette, further confirming the appropriate insertion of hACE2 with different strategies (D) Images of the healthy hACE2 mice generated from different strategies.

Four different targeting vectors were constructed as described below, cDNA encoding human *ACE2* was followed by the WPRE element, with the β‐globin either upstream or downstream of the cDNA sequence (Fig. 1A,B). The whole expressing cassette was inserted into the ATG site in the second exon of *mAce2* by homologous recombination using CRISPR‐Cas9 system. PGK‐Puro flanked by frt elements serves as a selection marker. The expression of inserted *hACE2* gene was driven by *mAce2* promoter. The sgRNA1 (5′‐tactgctcagtccctcaccgagg‐3′) and sgRNA2 (5′‐cttggcattttcctcggtgaggg‐3′) along with linearized donor vectors were transfected into mouse ESCs by electroporation. Sequences of β‐globin and optimized hACE2 can be found in File S1. Next, transfected cells were cultured under puromycin selection for 3 days, and the puromycin‐resistant colonies were kept for further validation. Only mouse ESCs with correct editing were used for tetraploid complementation assay.

### Tetraploid embryos/ES aggregation

Superovulated ICR females were mated with ICR males. E1.5 embryos at the two‐cell stage were flushed from the oviducts, placed between the electrodes of a 250 μm gap electrode chamber (BLS Ltd., Budapest, Hungary) containing 0.3 m Mannitol with 0.3% bovine serum albumin (Sigma‐Aldrich Inc., St. Louis, MO, USA), and fused with a cell fusion CF‐150/B apparatus (BLS Ltd.) with 0.5 mm Micro fusion slide (BTX‐450). An initial electrical field of 3 V was applied to the embryos, followed by one peak pulses of 50 V for 50 μS. The fused tetraploid embryos were cultured for 24 h toward the 4‐cell stage under the same conditions.

Mouse ESCs were aggregated and cultured with denuded 4‐cell stage mouse tetraploid embryos as reported with a slight modification [8]. In detail, aggregates of loosely connected ES cells (15–20 cells in each) were transferred into microdrops of KSOM medium with 10% FBS covered by mineral oil. Each aggregate was placed in a depression in the microdrop. Meanwhile, batches of 30–50 embryos were briefly incubated in acidified Tyrode's solution until dissolution of their zona pellucida. Two embryos were placed with one ES aggregate. All aggregates were assembled in this manner and cultured overnight at 37 °C, 5% CO_2_. After 24 h, 11–15 embryos were transferred into one uterine horn of a 2.5 days post‐coitum (DPC) pseudopregnant recipient. Mature CD‐1 females were used as pseudopregnant foster mothers with a weight of about 30 g.

### Mice infection

The hACE2 mice from different models were generated using mouse ESC‐based genomic editing combined with the tetraploid complementation assay. The hACE2 mice were then backcrossed with wild‐type Balb/c mice to obtain heterozygous hACE2 males and females. Heterozygous mice were subsequently mated to produce homozygous hACE2 offspring. The genotypes of all hACE2 mice were confirmed through Sanger sequencing. In total, 26 male and 6 female hACE2 F1 offspring were used for viral infection. Mice infection was performed following the protocol as reported before with slight modifications [9]. Briefly, hACE2 mice from three different models were intranasally infected with 1 × 10^4^ 50% tissue culture infective dose (TCID_50_) SARS‐CoV‐2 in 30 μL DMEM per mouse. The uninfected control mice were inoculated with DMEM only. Mice with undetectable viral gene expression in lung were regarded as uninfected mice and were excluded from the subsequent experiments. Mice were euthanized using isoflurane, followed by cervical dislocation at 3 days postinfection (dpi) per group, and lung tissues were collected for RNA samples.

### RNA isolation and real‐time quantitative PCR

Total RNA was extracted using a TRIzol‐based protocol. For real‐time quantitative PCR, cDNA was synthesized with ReverTra Ace (Toyobo, Shanghai, China) and oligo‐dT (Takara Bio, Beijing, China) and then analyzed by qPCR with Premix Ex Taq (Takara). Amplicons were normalized to the expression of *Gapdh*. All primers used in this study can be found in Table 1.

**Table 1:** Primers used in this study.

Primers	Sequences
hACE2‐qF	GGTCTTCAGTGCTCTCAG
hACE2‐qR	GCATTCTTGTGGATTATCTGG
opt‐hACE2‐qF	ACAATCCCCAAGAGTGCCTG
opt‐hACE2‐qR	CGTATTCCTCGTACAGGGGC
mGapdh‐qF	ACCTGCCAAGTATGATGAC
mGapdh‐qR	GGGAGTTGCTGTTGAAGT

### Transcriptome analysis

For polyA‐based mRNA‐seq, 1 μg total RNAs were used for library construction. mRNA was enriched and purified using Library Preparation VAHTSTM mRNA Capture Beads (Vazyme, NR401‐01, Nanjing, China). Next, purified mRNAs were fragmented, followed by cDNA synthesis, second‐strand synthesis, end repair, adaptor ligation, and amplification using VAHTS Universal V8 RNA‐seq Library Prep Kit for Illumina (Vazyme) and VAHTS DNA Clean Beads. Libraries were sequenced for an average of 20 million paired‐end reads.

The RNA‐seq data processing was performed as described below. To analyze the transcriptome changes, raw reads were first trimmed to remove adapter contamination and then aligned to both mouse mm10 genome reference and SARS‐CoV‐2 genome reference (NC_045512.2) using STAR (2.7.10a) with default settings [10]. FeatureCounts (2.0.1) was used for read assignment with the following parameters, −T 5 ‐g gene_name ‐p [11]. DEseq2 was used for data normalization, differential expression analysis, and data visualization [12]. Genes with *P*‐value < 0.05 and |log_2_(fold change)| > 1 were considered as differentially expressed genes and used for GO analysis.

### Western blotting

Mouse tissues were homogenized using Cell lysis buffer (20 mm Tris/HCl, pH 7.5, containing 150 mm NaCl, 2 mm DTT, 50% triglyceride, 100 mm EDTA, 1% SDS, 1% NP40 and 1% Triton X‐100) supplemented with protease inhibitor cocktails and phenylmethanesulfonyl fluoride (PMSF) (Beyotime, Shanghai, China). Western blots were performed using typical laboratory procedures with the following antibodies: anti‐ACE2 (ab108209, Abcam, Shanghai, China), anti‐GAPDH (MAB374, Millipore, Shanghai, China). Whole‐cell extracts were resolved by 12% SDS/PAGE, transferred to PVDF membranes, and probed with corresponding antibodies according to the manufacturer's recommendations (Cell Signaling Technology, Shanghai, China). All the uncropped blots can be found in the Fig. S1.

### H&E staining

Lung samples were fixed with 4% paraformaldehyde, followed by paraffin embedment. Fixed lung tissues were cut into 3.5‐μm sections for H&E staining. The whole procedure was done by servicebio technology in Wuhan. The images were collected using a Pannoramic MIDI system (3DHISTECH, Budapest, Hungary), and visualized using caseviewer (2.4). The lung injuries were evaluated and scored by servicebio technology following the International Harmonization of Nomenclature and Diagnostic Criteria for Lesions (INHAND) shown in Table 2. The detailed scoring can be found in the Table S1.

**Table 2:** Scoring criteria.

Score	Levels	Description
0	No changes	Under the research conditions, considering factors such as the animal's age, sex, and strain, the tissue is considered normal
1	Detectable	The observed changes have just exceeded the normal range
2	Mild	Lesions can be observed, but they are not yet severe
3	Moderate	The lesions are pronounced and are likely to become more severe
4	Marked	The lesions are extremely severe (they have affected the entire tissue or organ)

## Results

### Characterization of hACE2 mice from distinct mouse models

We have constructed four strategies to introduce different hACE2 cassettes into the *mAce2* locus in mouse genome (Fig. 1A–C). woodchuck hepatitis virus (WHP) posttranscriptional regulatory element (WPRE) is widely used to increase transgene expression by improving the 3′ RNA processing [13]. Thus, we employed WPRE downstream of the *hACE2* gene in the donor vector. It has been reported that the usage of the β‐globin intron, which often displays enhancer‐like function, could efficiently increase gene expression in eukaryotes [14, 15]. Therefore, we further improved our ACE2 expressing system by integrating β‐globin into the *hACE2* cassette. As enhancers can function either downstream or upstream of a gene to control its expression [16], we generated hACE2 mice with the β‐globin intron positioned either upstream or downstream of *hACE2* gene, namely β‐globin/hACE2 or hACE2/β‐globin mice (Fig. 1A–C). In parallel, we optimized hACE2 codons (opt‐hACE2) based on the preferential coding patterns observed in mice (Fig. 1A–C, and File S1). Mice from all the models are derived with no obvious developmental defects (Fig. 1D).

### Opt‐hACE2 mice exhibit more robust expression of hACE2

**Fig. 2:**
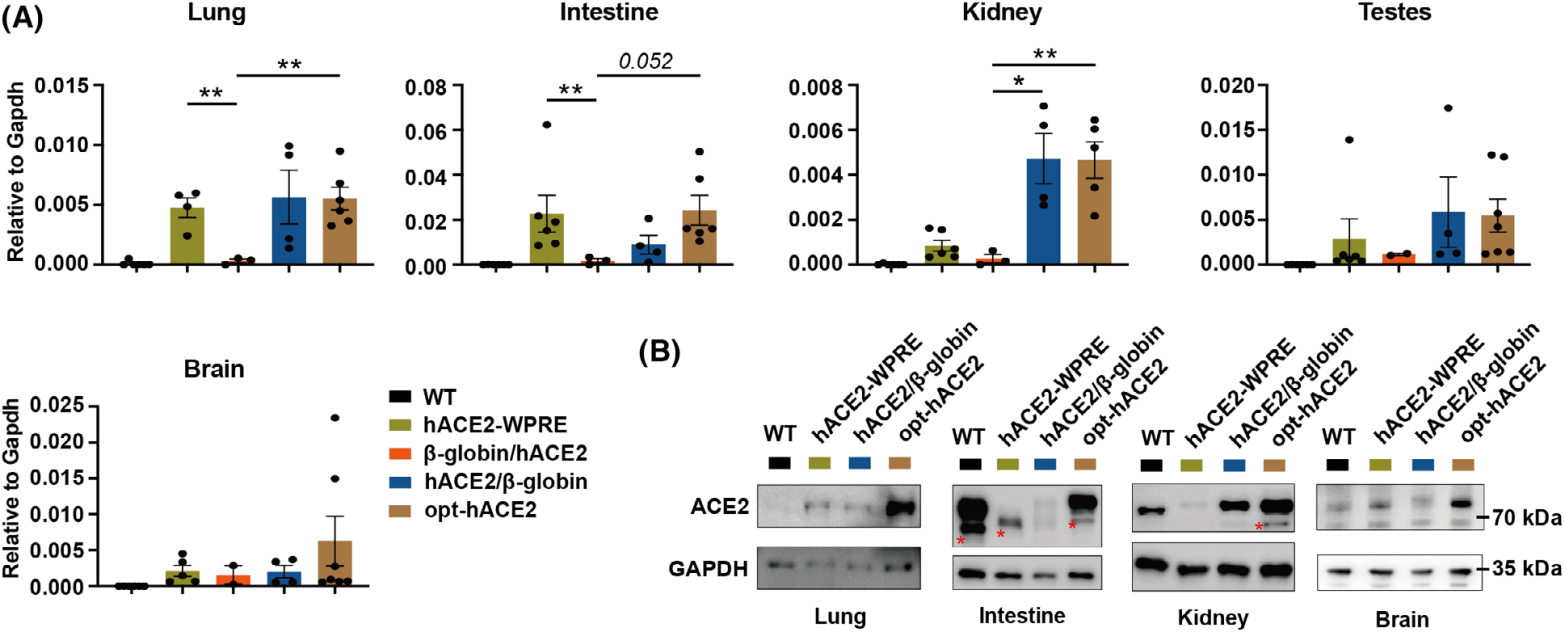
hACE2 expression in different tissues from four different hACE2 mouse models. (A) Quantification of mRNA levels of *hACE2* in lungs, kidneys, intestines, testes, and brains from wild‐type (*n* = 6), hACE2‐WPRE (*n* ≥ 4), β‐globin/hACE2 (*n* ≥ 2), hACE2/β‐globin (*n* = 4), and opt‐hACE2 (*n* ≥ 5) mouse models using RT‐qPCR. Data are shown as relative expression levels compared to *Gapdh*. Error bars, SEM; Student's *t*‐test, *P < 0.05, **P < 0.01. (B) Western blot analysis of hACE2 in lungs, kidneys, brains, and intestines from different hACE2 mouse models. * unspecific bands.

Next, we isolated multiple tissues including brains, testes, kidneys, intestines, and lungs from different mouse models and measured *hACE2* expression at mRNA levels using real‐time quantitative PCR (RT‐qPCR). In all tested tissues, we observed expression of *hACE2* mRNA in all four mouse models except for wild‐type mice (Fig. 2A). Comparison analysis across different hACE2 mouse models revealed an increased expression of *hACE2* mRNA in lungs, intestines, testes, and kidneys from hACE2/β‐globin mice, in which the β‐globin was positioned downstream of the WPRE element (Fig. 2A). Conversely, insertion of β‐globin upstream of hACE2 resulted in lower *hACE2* expression compared to WPRE only across nearly all tested tissues except the brains, suggesting a possible negative regulatory influence of upstream β‐globin on *hACE2* expression (Fig. 2A). Furthermore, optimization of hACE2 codons resulted in subtle changes on *hACE2* mRNA levels compared to the usage of standard codons (Fig. 2A), indicating a minimal effect of coding preference on transcription efficiency.

Next, we performed western blot to evaluate hACE2 protein levels in the lungs, kidneys, brains, and intestines. As β‐globin/hACE2 mice exhibited lower hACE2 expression compared to the other mouse models, we excluded these mice from the subsequent analysis and only focused on hACE2‐WPRE, hACE2/β‐globin, and opt‐hACE2 mice. A specific antibody targeting both human and mouse ACE2 proteins was utilized, with GAPDH serving as a loading control. Interestingly, despite the lower mRNA level of hACE2 in lungs of hACE2‐WPRE mice, we observed relatively comparable protein levels of hACE2 in the lungs between hACE2‐WPRE and hACE2/β‐globin mice, which are much higher than mACE2 from wild‐type mice (Fig. 2B). In kidney, hACE2‐WPRE resulted in a lower hACE2 protein level compared to both wild‐type mACE2 and hACE2/β‐globin protein (Fig. 2B). This is consistent with the observed transcription trend from qPCR results. In brains, comparable levels of hACE2 expression were observed among the lungs of wild‐type, hACE2‐WPRE, and hACE2/β‐globin mice. However, in these tissues, opt‐hACE2 mice exhibited highest hACE2 expression, suggesting enhanced translation efficiency using optimized codons (Fig. 2B). In intestines, we only detected hACE2 protein from opt‐hACE2 mice with similar level comparing to that from wild‐type mice (Fig. 2B). Altogether, our findings demonstrated that incorporation of β‐globin downstream of *hACE2* alongside optimized hACE2 codons sufficiently increase hACE2 expression across distinct mouse tissues.

### Opt‐hACE2 mice display a more pronounced immune response upon viral infection

**Fig. 3:**
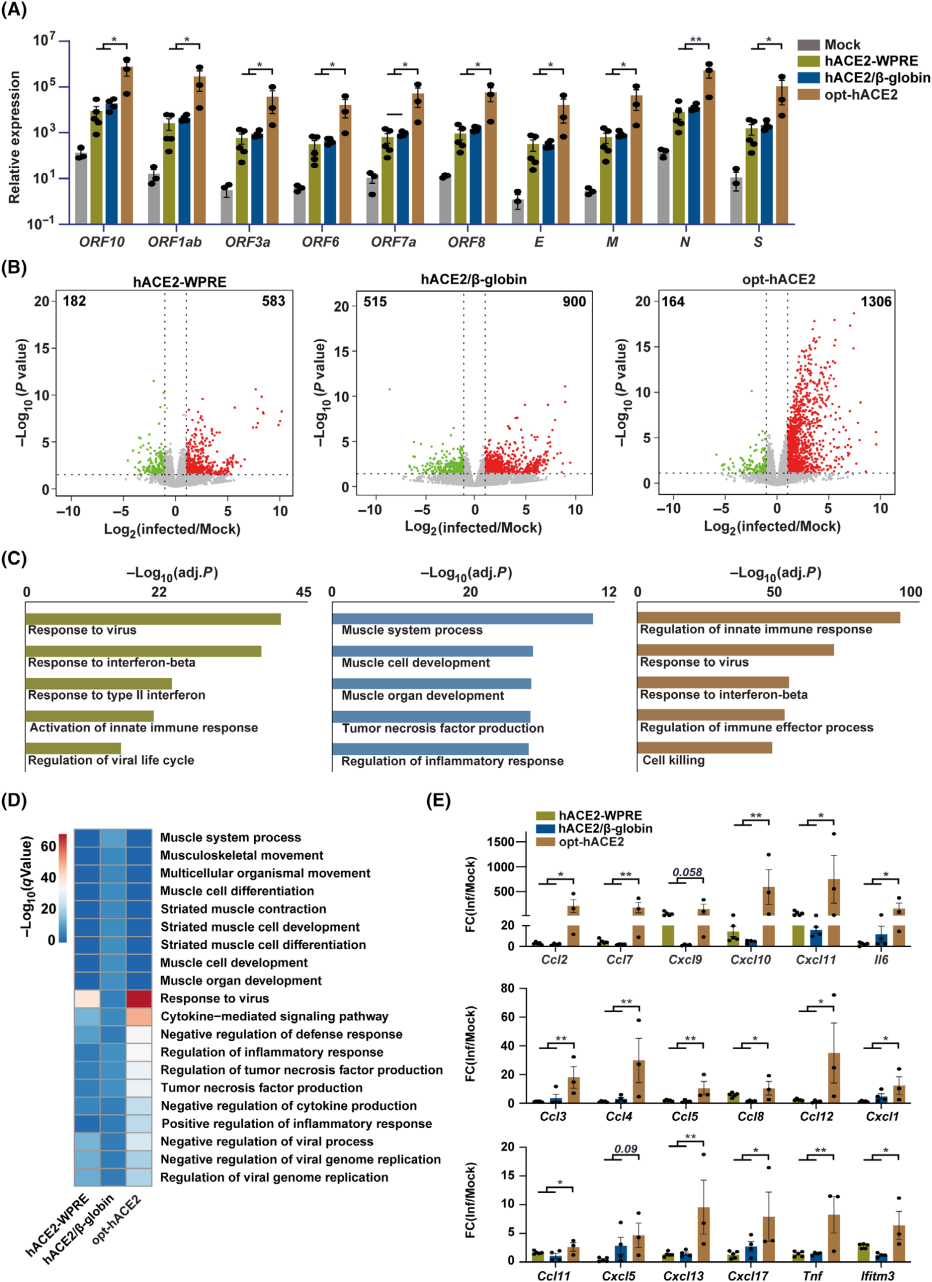
Viral infection results in COVID‐19 phenotypes in all tested hACE2 mouse models. (A) Expression levels of viral genes in the lungs of infected hACE2 mice from mock (*n* = 3), infected hACE2‐WPRE (*n* = 5), hACE2/β‐globin (*n* = 4), and opt‐hACE2 (*n* = 3) mice. *ORF3a*, *E*, and *S* expression levels are detectable in only 2 out of 3 mock replicates. Error bars, SEM from at least 3 biological replicates. Student's *t*‐test, *P < 0.05, **P < 0.01. (B) Volcano plots showing transcriptome changes in the lungs between uninfected and infected hACE2 mice from different mouse models. Significantly differentially expressed genes (|log_2_(fold change)| > 1, *P*‐value < 0.05) are highlighted in green (upregulated in control) and red (upregulated in the infected mice). (C) GO analysis of activated genes in the lungs of hACE2 mice from different models upon viral challenging. (D) Heatmap showing the enrichment of GO terms from different infected hACE2 mice. Data were shown as −log_10_(*q*Value). (E) Bar plots showing the expression levels of genes involved in immune response pathway in the lungs of infected hACE2‐WPRE (*n* = 5), hACE2/β‐globin (*n* = 4), and opt‐hACE2 (*n* = 3) mice. Data from A and E were shown as normalized expression representing normalized read counts from RNA‐seq data. Error bars, SEM from at least 3 biological replicates. Student's *t*‐test, *P < 0.05, **P < 0.01.

To further explore the potential use of our mouse models for COVID‐19 study, we intranasally infected our hACE2 mice with 1 × 10^4^ 50% tissue culture infective dose (TCID_50_) of SARS‐CoV‐2 from Wuhan (IVCAS 6.7512). Lung samples were collected at 3 days postinfection (dpi) for transcriptomic and histological analyses. Overall, no clear differences were observed between infected male and female mice within each model. Consistent with the hACE2 expression pattern, opt‐hACE2 mice exhibited higher expression of SARS‐CoV‐2 genes including *E*, *M*, *N*, *S*, and *ORF* genes in the lungs compared to other mouse models, suggesting possibly greater viral load in the lungs of opt‐hACE2 mice (Fig. 3A).

Transcriptome analysis of the hosts identified in total 386, 1415, and 1283 significantly differentially expressed genes (|log_2_FoldChnage| > 1, *P* < 0.05) between infected and uninfected mice from each mouse models, respectively (Fig. 3B). Gene ontology enrichment analysis of activated genes upon viral infection revealed strong activation of immune response associated pathways including defense response to virus, regulation of innate immune response, response to interferon‐beta, regulation of inflammatory response, and tumor necrosis factor production in all the mouse models (Fig. 3C,D), indicating an overall successful infection of SARS‐CoV‐2 on all tested mouse models. Interestingly, besides the active immune pathways shared by all the mouse models, we also observed biological processes, for instance muscle development‐related pathways, were significantly perturbated in the lungs of hACE2/β‐globin mice. Next, we compared the transcriptional alterations of the infected mice between different models. We focused on the genes that are differentially expressed compared to the uninfected controls in each group. A comparison of dysregulated pathways across different mouse models revealed a more significant enrichment of immune pathways in opt‐hACE2 mice (Fig. 3D). In agreement with GO analysis, the expression of immune‐related genes such as *Ccl* genes, *Cxcl* genes, *Il6*, *Tnf*, and *Ifitm3* were further increased in the lungs of opt‐hACE2 mice after viral infection (Fig. 3E), highlighting a more pronounced immune response in these mice compared to the other mouse models.

### Infection with SARS‐CoV‐2 led to pathological phenotypes of COVID‐19 in hACE2 mice from different models

**Fig. 4:**
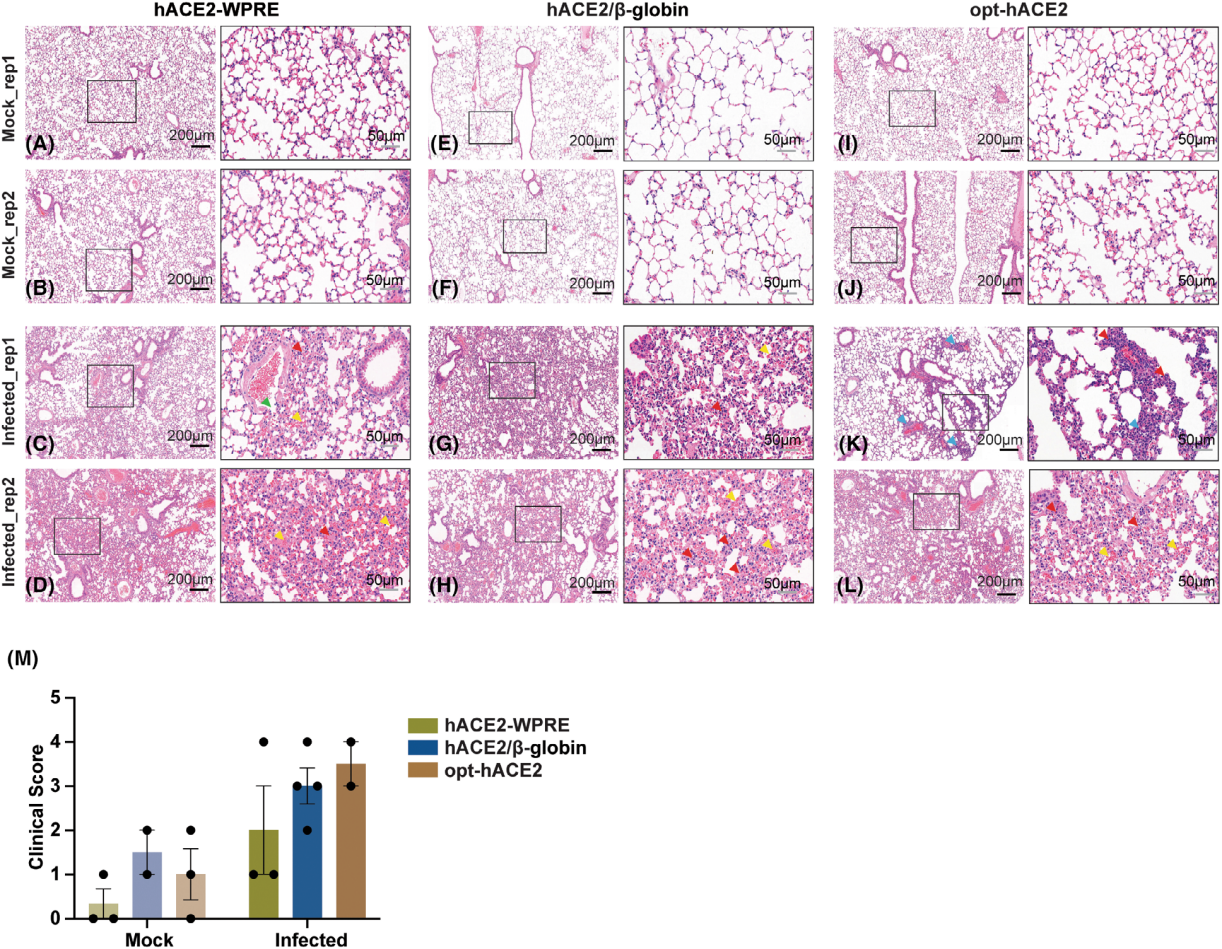
Histological changes revealed a more active immune response in opt‐hACE2 mice. Lung tissues of hACE2 mice from different mouse models challenged by SARS‐CoV‐2 at 3 days postinfection were stained with H&E. Lesions are not observed in uninfected mice (A–B, E–F, I–J). In lungs of infected hACE2‐WPRE and hACE2/β‐globin mice, staining with H&E shows thickened alveolar walls and pulmonary hemorrhage (yellow arrows) along with mild inflammatory cell infiltration (C–D, G–H). In opt‐hACE2 mice, lung tissues show thickened alveolar walls, mild pulmonary hemorrhage, and more severe inflammatory cell infiltration after rival infection. Red arrows, thickened alveolar walls with immune cell infiltration; yellow arrows, hemorrhage; green arrows, vascular leakage. Black scale bar = 200 μm, and green scale bar = 50 μm. (M) Quantitative scoring of pathology of lung tissues from mock and infected hACE2‐WPRE (3 vs 3), hACE2/β‐globin (2 vs 4), and opt‐hACE2 (3 vs 2) mice. Error bars indicate the standard error of mean.

To further elucidate whether these infected mice developed pneumonia, lung tissues were collected at 3 dpi for hematoxylin and eosin (H&E) staining. Histopathological analysis revealed similar degrees of lung damage among the infected hACE2‐WPRE, hACE2/β‐globin, and opt‐hACE2 mice. Compared to mock‐infected mice, several histological changes including slightly thickened alveolar walls, pulmonary hemorrhage in the alveoli, and vascular leakage were observed in lungs from hACE2‐WPRE mice after viral infection (Fig. 4A–D). Similarly, hACE2/β‐globin mice displayed comparable levels of hemorrhage and thickened alveolar walls in peri‐bronchial and pulmonary alveolar regions, indicating similar pathogenic outcomes in infected lungs between these two mouse models (Fig. 4E–H). However, in the opt‐hACE2 mice, which express higher levels of hACE2 and harbour more viral loads, microscopy revealed severe damages of alveolar structure, thickened alveolar walls with mixed inflammatory cell infiltration, and hemorrhage in the lungs after viral infection (Fig. 4I–L), suggesting a more pronounced infectious effect compared to the other models. The quantitative pathological scoring which showed similar trends further confirm the hematoxylin–eosin (H&E) pathology observation (Fig. 4M and File S1). Altogether, these findings demonstrate that our mouse models exhibit multiple COVID‐19 features, with opt‐hACE2 mice showing a stronger immune response and more severe pathogenesis upon viral challenge.

## Discussion

In our study, we have comprehensively compared various hACE2 mouse models generated with different strategies to enhance hACE2 expression. These strategies included the use of WPRE, β‐globin, and coding optimization. β‐globin is widely used in combination with the CMV promoter and chicken β‐actin, known as CAG hybrid promoter. Extensive studies have utilized the CAG promoter to express transgenes in mESCs and during mESC differentiation [15, 16, 17], indicating a positive role of β‐globin in transcription regulation. However, our findings suggest that placing β‐globin upstream of *hACE2* may negatively regulate its expression, highlighting the critical influence of relative positioning on its regulatory function. Variations in hACE2 expression across different tissues were observed in our mouse models, indicating that additional layers of transcription regulatory machinery exist to control hACE2 expression beyond the WPRE and β‐globin elements. Local chromatin states are crucial in regulating gene transcription [18]. Therefore, we hypothesize that chromatin dynamics at the *Ace2* locus between different tissues may contribute to the observed diversity in hACE2 expression. Further investigation on how exogenous gene regulatory elements interact with the endogenous ones warrants attention and future research.

Although coding optimization does not affect hACE2 transcription levels, it significantly enhances the translation efficiency. Consistent with hACE2 expression, SARS‐CoV‐2 infection on opt‐hACE2 mice resulted in increased viral entry and active immune response in the lungs compared to other mouse models. Our results align with recent studies demonstrating a positive correlation between hACE2 expression and viral loads [19]. Severe COVID‐19 often leads to pneumonia, alongside pulmonary bronchial and alveolar epithelial cell dysfunction, which is associated with respiratory failure and high mortality. Notably, none of our hACE2 mice died during viral infection. Given that aging is a high‐risk factor for severe COVID‐19 [20], we propose that future trials using aged opt‐hACE2 mice for SARS‐CoV‐2 could better phenocopy severe COVID‐19 in humans. Besides lungs, SARS‐CoV‐2 infection could affect other tissues such as brains, hearts, intestines, and blood vessels [21, 22]. These impacts might cause tissue‐specific dysfunction, potentially leading to a health problem as well. Therefore, further investigation could focus on the changes in other tissues, which would provide valuable insights into the utility of our hACE2 mouse models. The original SARS‐CoV‐2 strain was used in the current study. By now, multiple prominent SARS‐CoV‐2 variants exist including Alpha, Beta, Delta, and Omicron, which remain to be well studied [23, 24]. It would be of great significance to know how our hACE2 mouse models respond to different SARS‐CoV‐2 variants. We believe this would provide valuable insights and could potentially broaden the application of the model in future SARS‐CoV studies. Overall, our study has generated diverse hACE2 models with various strategies and evaluated their potential utility in COVID‐19 research.

In conclusion, this study provides valuable insights into optimizing hACE2 expression strategies in mice, highlighting that positioning the β‐globin intron appropriately and optimizing coding preference enhance hACE2 expression when inserted at *Ace2* locus in mice. Furthermore, our work has evaluated the potential usage of these hACE2 mice in COVID‐19 studies, demonstrating that opt‐hACE2 mice could serve as a valuable model for SARS‐CoV‐2 studies.

## Conflict of interest

Author Wu Guangming is currently running a company called MingCeler Biotech Co Lt, which may be affected by the research reported in the enclosed paper. The other authors have no competing interests.

### Peer review

The peer review history for this article is available at https://www.webofscience.com/api/gateway/wos/peer‐review/10.1002/2211‐5463.13934.

## Author contributions

SJ, WH, and WG conceived the study. SJ, CS, and WH designed the experiments, interpreted the data, and prepared the illustrations. QX, MR, and SJ prepared the animal models. SJ, CS, and WH contributed to the data analysis. LM and LH performed the viral infection. SJ, LM, WH, and CJ supervised experiments. SZ, JG, CJ, and WG provided resources. WH wrote the main manuscript text. All authors commented and proofread the manuscript.

## Supporting information


**Fig. S1.** Uncropped blots for data show in Fig. 2B.
**Table S1.** The detailed lung histopathology scores for each sample.
**File S1.** DNA sequences of β‐globin and Optimized *hACE2*.

## Data Availability

The data that support the findings of this study are openly available in NCBI's Gene Expression Omnibus and are accessible through https://www.ncbi.nlm.nih.gov/geo/query/acc.cgi?acc=[GSE271163], with GEO Series accession number [GSE271163].
