# Supplementary material for: Enhancing human ACE2 expression in mouse models to improve COVID‐19 research
Source: FEBS Open Bio. 2024 Dec 29;15(2):324–34. doi: 10.1002/2211-5463.13934 (PMC11788745; doi:10.1002/2211-5463.13934)
Supplement: Supplementary file 1 — Fig. S1. Uncropped blots for data show in Fig. 2B. Table S1. The detailed lung histopathology scores for each sample. File S1. DNA sequences of β‐globin and Optimized hACE2. [file FEB4-15-324-s001.docx]

**Supporting Information**

Fig. S1. Uncropped blots for data show in Figure 2B.

Table S1. The detailed lung histopathology scores for each sample.

File S1. DNA sequences of β-globin and Optimized *hACE2*.

**
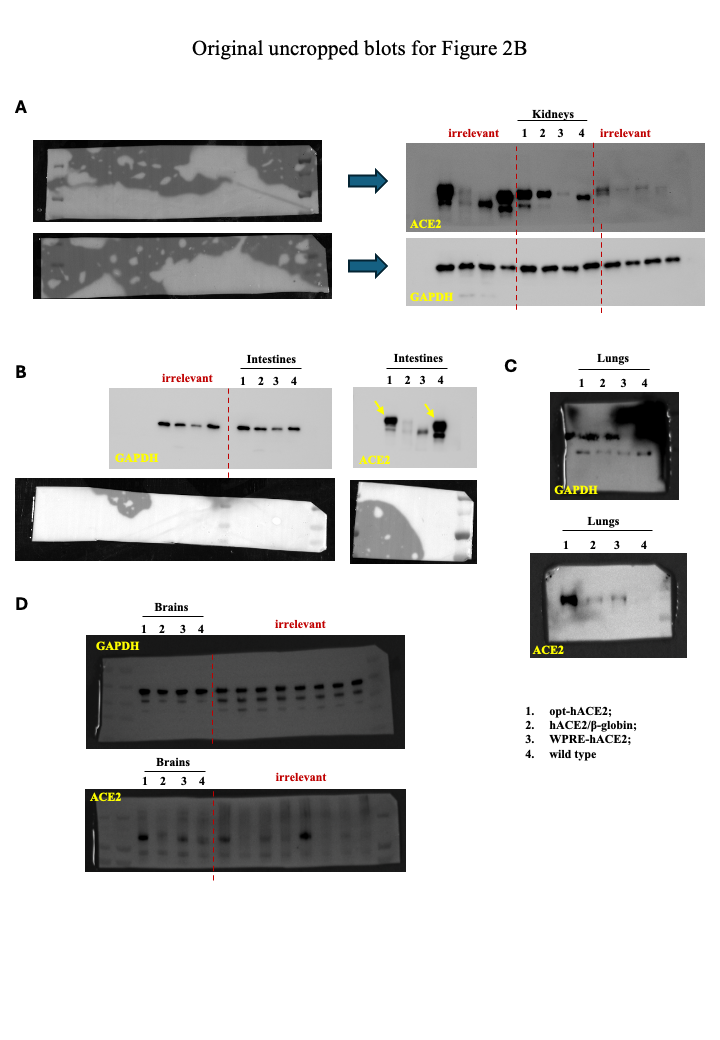
**

Fig. S1. Uncropped blots for data show in Figure 2B. The Original uncropped blots of ACE2 and GAPDH in kidneys (A), intestines (B), lungs (C), and brains (D) for Figure 2B.

Supplementary table 1. The detailed lung histopathology scores for each sample.

| Samples | thickened alveolar walls | inflammatory cell infiltration | congestion | hemorrhage |
| --- | --- | --- | --- | --- |
| hACE2-WPRE_Mock_rep1 | 0 | 0 | 0 | 0 |
| hACE2-WPRE_Mock_rep2 | 0 | 0 | 1 | 0 |
| hACE2-WPRE_Mock_rep3 | 0 | 0 | 0 | 0 |
| hACE2/β-globin_mock_rep1 | 1 | 0 | 0 | 1 |
| hACE2/β-globin_mock_rep2 | 0 | 0 | 1 | 0 |
| opt-hACE2_Mock_rep1 | 0 | 0 | 1 | 0 |
| opt-hACE2_Mock_rep2 | 0 | 0 | 1 | 1 |
| opt-hACE2_Mock_rep3 | 0 | 0 | 0 | 0 |
| hACE2-WPRE_inf_rep1 | 0 | 0 | 1 | 0 |
| hACE2-WPRE_inf_rep2 | 0 | 0 | 1 | 0 |
| hACE2-WPRE_inf_rep3 | 1 | 1 | 2 | 0 |
| hACE2/β-globin_inf_rep1 | 1 | 1 | 1 | 0 |
| hACE2/β-globin_inf_rep2 | 0 | 1 | 1 | 0 |
| hACE2/β-globin_inf_rep3 | 1 | 1 | 1 | 0 |
| hACE2/β-globin_inf_rep4 | 1 | 1 | 2 | 0 |
| opt-hACE2_inf_rep1 | 0 | 1 | 2 | 0 |
| opt-hACE2_inf_rep2 | 1 | 1 | 2 | 0 |

File S1. DNA sequences of β-globin and Optimized hACE2.

β-globin sequence:

GTTAACGTGAGTTTGGGGACCCTTGATTGTTCTTTCTTTTTCGCTATTGTAAAATTCATGTTATATGGAGGGGGCAAAGTTTTCAGGGTGTTGTTTAGAATGGGAAGATGTCCCTTGTATCACCATGGACCCTCATGATAATTTTGTTTCTTTCACTTTCTACTCTGTTGACAACCATTGTCTCCTCTTATTTTCTTTTCATTTTCTGTAACTTTTTCGTTAAACTTTAGCTTGCATTTGTAACGAATTTTTAAATTCACTTTTGTTTATTTGTCAGATTGTAAGTACTTTCTCTAATCACTTTTTTTTCAAGGCAATCAGGGTATATTATATTGTACTTCAGCACAGTTTTAGAGAACAATTGTTATAATTAAATGATAAGGTAGAATATTTCTGCATATAAATTCTGGCTGGCGTGGAAATATTCTTATTGGTAGAAACAACTACATCCTGGTCATCATCCTGCCTTTCTCTTTATGGTTACAATGATATACACTGTTTGAGATGAGGATAAAATACTCTGAGTCCAAACCGGGCCCCTCTGCTAACCATGTTCATGCCTTCTTCTTTTTCCTACAG

Optimized hACE2 sequence:

ATGTCTAGCAGCTCTTGGCTGCTGCTGTCTCTGGTGGCTGTGACAGCCGCTCAGAGCACCATTGAGGAACAGGCCAAGACCTTCCTGGACAAGTTCAACCACGAGGCCGAGGACCTGTTCTACCAGTCTAGCCTGGCCAGCTGGAACTACAACACCAACATCACCGAAGAGAACGTGCAGAACATGAACAACGCCGGCGACAAGTGGAGCGCCTTCCTGAAAGAGCAGAGCACACTGGCCCAGATGTACCCTCTGCAAGAGATCCAGAACCTGACCGTGAAGCTCCAGCTGCAGGCCCTCCAGCAGAATGGAAGCTCTGTGCTGAGCGAGGACAAGAGCAAGCGGCTGAACACCATCCTGAATACCATGAGCACCATCTACAGCACCGGCAAAGTGTGCAACCCCGACAATCCCCAAGAGTGCCTGCTGCTGGAACCCGGCCTGAATGAGATCATGGCCAACAGCCTGGACTACAACGAGAGACTGTGGGCCTGGGAGTCTTGGAGAAGCGAAGTGGGAAAGCAGCTGCGGCCCCTGTACGAGGAATACGTGGTGCTGAAGAACGAGATGGCCAGAGCCAACCACTACGAGGACTACGGCGACTATTGGAGAGGCGACTACGAAGTGAATGGCGTGGACGGCTACGACTACAGCAGAGGCCAGCTGATCGAGGACGTGGAACACACCTTCGAGGAAATCAAGCCTCTGTACGAGCATCTGCACGCCTACGTGCGGGCCAAGCTGATGAATGCTTACCCCAGCTACATCAGCCCCATCGGCTGTCTGCCTGCTCATCTGCTGGGAGACATGTGGGGCAGATTCTGGACCAACCTGTACAGCCTGACAGTGCCCTTCGGCCAGAAACCTAACATCGACGTGACCGACGCCATGGTGGATCAGGCTTGGGATGCCCAGCGGATCTTCAAAGAGGCCGAGAAGTTCTTCGTGTCCGTGGGCCTGCCTAATATGACCCAAGGCTTCTGGGAGAACTCCATGCTGACAGACCCCGGCAATGTGCAGAAAGCCGTGTGTCATCCTACCGCCTGGGATCTCGGCAAGGGCGACTTCAGGATCCTTATGTGCACCAAAGTGACGATGGACGACTTCCTGACAGCCCACCACGAGATGGGCCACATCCAGTACGATATGGCCTACGCCGCTCAGCCCTTCCTGCTGAGAAATGGCGCCAATGAGGGCTTCCACGAAGCCGTGGGAGAGATCATGAGCCTGTCTGCCGCCACACCTAAGCACCTGAAGTCTATCGGACTGCTGAGCCCCGACTTCCAAGAGGACAACGAGACAGAGATCAACTTCCTGCTCAAGCAGGCCCTGACCATCGTGGGCACACTGCCCTTTACCTACATGCTGGAAAAGTGGCGGTGGATGGTCTTTAAGGGCGAGATCCCCAAGGACCAGTGGATGAAGAAATGGTGGGAGATGAAGCGCGAGATCGTGGGCGTTGTGGAACCTGTGCCTCACGACGAGACATACTGCGATCCTGCCAGCCTGTTTCACGTGTCCAACGACTACTCCTTCATCCGGTACTACACCCGGACACTGTACCAGTTCCAGTTTCAAGAGGCTCTGTGCCAGGCCGCCAAGCACGAAGGACCTCTGCACAAGTGCGACATCAGCAACTCTACAGAGGCCGGACAGAAACTGTTCAACATGCTGCGGCTGGGCAAGAGCGAGCCTTGGACACTGGCTCTGGAAAATGTCGTGGGCGCCAAGAATATGAACGTGCGGCCACTGCTGAACTACTTCGAGCCCCTGTTCACCTGGCTGAAGGACCAGAACAAGAACAGCTTCGTCGGCTGGTCCACCGATTGGAGCCCTTACGCCGACCAGAGCATCAAAGTGCGGATCAGCCTGAAAAGCGCCCTGGGCGATAAGGCCTATGAGTGGAACGACAATGAGATGTACCTGTTCCGGTCCAGCGTGGCCTATGCTATGCGGCAGTACTTTCTGAAAGTCAAGAACCAGATGATCCTGTTCGGCGAAGAGGATGTGCGCGTGGCCAACCTGAAGCCTCGGATCAGCTTCAACTTCTTCGTGACTGCCCCTAAGAACGTGTCCGACATCATCCCCAGAACCGAGGTGGAAAAGGCCATCAGAATGAGCAGAAGCCGGATCAACGACGCCTTCCGGCTGAACGACAACTCCCTGGAATTCCTGGGCATTCAGCCCACACTGGGCCCTCCAAATCAGCCTCCTGTGTCCATCTGGCTGATCGTGTTTGGCGTGGTCATGGGCGTGATCGTCGTGGGCATCGTGATCCTGATCTTCACCGGCATCCGCGACCGGAAGAAGAAGAACAAGGCCAGAAGCGGCGAGAACCCCTACGCCAGCATCGATATCAGCAAAGGCGAGAACAACCCCGGCTTCCAGAACACCGATGACGTGCAGACCAGCTTCTGA
